# A Fully Integrated Assay Panel for Early Drug Metabolism and Pharmacokinetics Profiling

**DOI:** 10.1089/adt.2020.970

**Published:** 2020-05-20

**Authors:** Johan Wernevik, Fredrik Bergström, Anna Novén, Johan Hulthe, Linda Fredlund, Dan Addison, Jan Holmgren, Per-Erik Strömstedt, Erika Rehnström, Thomas Lundbäck

**Affiliations:** ^1^ Mechanistic Biology & Profiling, Discovery Sciences, R&D, AstraZeneca, Gothenburg, Sweden.; ^2^ DMPK, Early CVRM, BioPharmaceuticals R&D, AstraZeneca, Gothenburg, Sweden.; ^3^ Sample Management, Discovery Sciences, R&D, AstraZeneca, Cambridge, United Kingdom.; ^4^ Sample Management, Discovery Sciences, R&D, AstraZeneca, Gothenburg, Sweden.; ^5^ Clinical Sampling & Alliances, Precision Medicine, AstraZeneca, Gothenburg, Sweden.

**Keywords:** mass spectrometry, automation, DMPK, profiling, assay panels

## Abstract

Evaluation and optimization of physicochemical and metabolic properties of compounds are a crucial component of the drug development process. Continuous access to this information during the design-make-test-analysis cycle enables identification of chemical entities with suitable properties for efficient project progression. In this study, we describe an integrated and automated assay panel (DMPK Wave 1) that informs weekly on lipophilicity, solubility, human plasma protein binding, and metabolic stability in rat hepatocytes and human liver microsomes. All assays are running in 96-well format with ultraperformance liquid chromatography–mass spectrometry (MS)/MS as read-out. A streamlined overall workflow has been developed by optimizing all parts of the process, including shipping of compounds between sites, use of fit-for-purpose equipment and information systems, and technology for compound requesting, data analysis, and reporting. As a result, lead times can be achieved that well match project demands across sites independently of where compounds are synthesized. This robust screening strategy is run on a weekly basis and enables optimization of structure-activity relationships in parallel with DMPK properties to allow efficient and informed decision making.

## Introduction

Reduction of attrition rates in clinical studies is a critical objective for improving R&D efficiency.^1^ Hence, significant investments are going into the optimization of compounds toward candidate drugs, such that their properties allow adequate testing of clinical hypotheses. This is achieved through iterative design-make-test-analyze (DMTA) cycles, where compounds are characterized for their impact on the pharmacology of interest as well as aspects that control drug metabolism and pharmacokinetic (DMPK) properties.^2–4^

Historically DMPK assays were run on selected compounds as dictated by low-throughput assays. As these assays were transferred to 96-well formats the capacity grew to support bespoke orders placed by DMPK project leads, with waves of assay panels of increasing complexity supporting projects based on maturity and needs.^5^ In 2012, AstraZeneca initiated a strategic initiative, in which part of the synthetic chemistry and DMPK work was outsourced, with careful efforts to ensure concordance between tests and results between sites.^6^

This on-going research exchange includes the DMPK Wave 1 panel of assays, which report on physicochemical properties such as lipophilicity (logD7.4) and solubility (Sol), plasma protein binding (PPB), and two metabolic stability assays in human liver microsomes (HLMs) (Mics) and rat hepatocytes (Heps), respectively. As of today, this panel is run on a weekly basis on all synthesized compounds across AstraZeneca to ensure maximal integration of DMPK aspects already at early stages of the DMTA cycle.

**Fig. 1.**
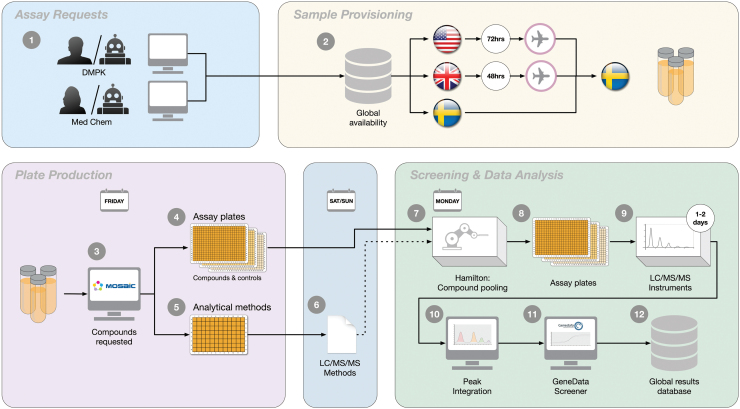
Schematic illustration of the integrated process for the DMPK Wave 1 panel in Gothenburg. Assay requests are made automatically or manually by DMPK or Medicinal Chemistry leads (1), triggering provisioning of samples via Sample Management to Gothenburg (2). Shipping complies with a 48 h time frame from the United Kingdom and a 72 h time frame from the United States. Once available, compounds are ordered to test, with one set of plates for the panel assays (3) and another set of plates for establishment of analytical methods (4). Ordering is integrated with compound pooling scripts based on their monoisotopic mass to reduce analysis time on the MS instrumentation (5). Each assay also includes dedicated assay performance controls (6). LC/MS/MS methods are created over the weekend (7) and stored and shared between instruments. The assays start weekly and run between 1 and 2 days (8), with identification and integration of chromatographic peaks in dedicated software (9). Integrated data are imported into GeneData Screener for further quality control and calculations (10) and finally made available globally by submission into an internal database (11). DMPK, drug metabolism and pharmacokinetic; LC, liquid chromatography; MS, mass spectrometry. Color images are available online.

Some of the individual assays within the DMPK Wave 1 panel have been previously described,^7,8^ while extensive efforts have subsequently been completed to integrate the assays in a fully automated workflow. In addition, these assays have over time been consolidated to one of our R&D facilities in Gothenburg and a partner site (Pharmaron). The assay workflow is schematically illustrated in *Figure 1* and includes compound requests, shipping of compounds to Gothenburg if not already available, establishment of mass spectrometry (MS)-based analysis methods for the individual compounds, completion of the five assays, and reporting through Genedata Screener to our internal database.

In this study, we describe the integrated workflow, including detailed descriptions of test methods and reporting routines. Finally, we provide assay metrics data after 6 years of operation and detail a case study where the assay panel had a significant impact on compound optimization.

## Materials and Methods

### Reagents and Consumables

All compounds and quality controls (QCs) were obtained internally from Sample Management facilities as 10 mM stock solutions in dimethyl sulfoxide (DMSO). DMSO was from Sigma-Aldrich (D2650), formic acid (FA) was of p.a. quality (*e.g.*, Merck, Germany), acetonitrile (AcN) was of high performance liquid chromatography (HPLC) grade (*e.g.*, Rathburn, Scotland), water was from a Milli-Q system (Millipore), and 1-Octanol was from Sigma-Aldrich (293245). Pipette tips for the Hamilton STARplus™ liquid handling robot were ZT-100-L-R (301-12-101; Genuine Axygen) and ZT-200-L-R (301-12-251; Genuine Axygen). Microplates and accessories were Nunc 2 mL polypropylene (PP)-based deep well plate (278752; Thermo Fisher Scientific), Nunc 1 mL 96-deep well PP Plate Natural (260252; Thermo Fisher Scientific), conical Nunc plates 0.45 mL/well (249944; Thermo Fisher Scientific), Nunc microplate lids (263339; Thermo Fisher Scientific), Nunc 96-well cap natural (276002; Thermo Fisher Scientific).

### Assay Automation

Three Hamilton Microlab STARplus robots (Hamilton Robotics, USA) controlled with Hamilton's Venus Software have been setup in an identical configuration to support all five DMPK Wave 1 assays. The setup of the robots consists of a 96-well pipetting head, an eight-channel pipetting arm, both capable of moving plates or lids. Several carriers that are used for the different assays are available and interchangeable between robots. Each robot is equipped with three Hamilton heated shakers that are temperature controlled and have a custom holder for the plate types used for the reaction plates in the metabolic stability assays. This minimizes potential edge effects due to temperature differences between ambient and incubation temperature. In addition, there are 10 temperature-controlled positions (water bath) with custom adapters for the plates.

### Ultraperformance Liquid Chromatography/MS

Sensitive, selective, and rapid methods for the analysis of samples from the assays were based on ultraperformance liquid chromatography (UPLC-MS)/MS. The chromatographic separations are performed on a Waters Acquity UPLC HSS T3 column packed with 1.8 μm particles, 2.1 × 30 mm, with a short gradient elution for samples from metabolic incubations. Here, the mobile phases consist of A (water with 0.1% FA) and B (AcN with 0.1% FA). The liquid chromatography (LC) gradient profile is as follows: 0.2% B during 0 to 0.1 min, a linear increase to 95% B during 0.1 to 0.7 min, hold at 95% B during 0.7 to 1.0 min, and then back to 0.2% B from 1.01 to 1.1 min. Analysis of samples from the logD7.4, Sol, and PPB assays, where up to 13 different analytes are analyzed, was based on a Waters Acquity UPLC HSS T3 column packed with 1.8 μm particles, 2.1 × 50 mm, and a 2 min gradient elution.

The LC gradient profile for these assays is as follows: 0.2% B during 0 to 0.3 min, a linear increase to 95% B during 0.3 to 1.3 min, hold at 95% B during 1.3 to 1.8 min, and then back to 0.2% B from 1.8 to 1.81 min. The flow rate is 1 mL/min with a total run time of 1.5 and 2.9 min, respectively, per sample. Detection is achieved by the multiple reactions monitoring (MRM) of the transitions determined in the optimization process by using QuanOptimize (Waters, Manchester, United Kingdom). LC-MS is carried out on a Waters Xevo TQS triple quadrupole equipped with a Waters Acquity UPLC interface, allowing switching between positive and negative ionization modes (Waters).

### MS/MS Optimization

All compounds to be screened are delivered from in-house Sample Management as 10 mM DMSO solutions in 96-well format. The compounds are diluted in a 1:2 AcN/water (0.1% FA) solution. We consider 1 μM optimal for optimization on the Waters Xevo TQS instrument and 5 μM for using the Waters TQD instrument. The QuanOptimize software (Waters) is used as a high-throughput tool for automated method development and batch processing of quantitative bioassays. The QuanOptimize routines can ramp and optimize cone voltage and collision energy. The cone voltage is set to 35 eV for all compounds and only the collision energy is optimized and ramped both in positive and negative mode between 10 and 50 eV in steps of six.

The software identifies the most intense fragment and the corresponding collision energy and creates an MRM transition, which is stored in an MS/MS library. The MS/MS library is shared between the LC-MS/MS instruments. QuanOptimize is also used for creating the sample list by generating MS/MS files from the MS/MS library combining up to 13 different MRM transitions, LC file, MS/MS tune file, and creating quantification methods used for peak integration, calibration, and quantification.

### Pooling

Pooling of compounds is used as a strategy to increase assay capacity and reduce analysis time in the mass spectrometers. All assays have dedicated excel-based macros to enable optimal pooling. In the logD7.4 and PPB assays, pooling of compounds occurs at assay start, whereas in the Sol, Mics, and Heps assays pooling is achieved after the experimental procedures, but before analysis. We recognize that there is a risk of interactions and/or interferences between compounds in the same pools, especially in the logD7.4 and PPB assays as pooling is done already in the physically/biologically relevant assay step. These concerns are addressed in the main text for each of these assays, each of which is qualified by the illustration of test results from two separate test occasions (and thus different pools) and between AstraZeneca and the Contract Research Organization (CRO) (also representing different pools).

Up to 13 compounds are pooled, depending on the assay. To avoid coelution, the pooling is designed by sorting the compounds based on molecular weight and then the compound with lowest mass will be placed in pool 1 and the second lowest in pool 2, and after 10–13 pools, there is enough difference to avoid mass conflicts in the first quadrupole of the mass spectrometer. Fast scanning triple quadrupole mass spectrometers are used to get ten data points over the chromatographic peak that is about 1.5 s wide at baseline. Positive and negative MS/MS methods are often combined. UPLC is used to increase selectivity and sensitivity.

### Statistical Analysis

All assays include three or more QCs in each run and two statistical tools are used to monitor performance of the control compounds. First, the in-house developed “Manhattan tool”, which allows monitoring of the performance of the control compounds by plotting the data against the date of the run.^6^ Acceptance criteria for control compounds are defined for each control in all the assays and a statistical change in the performance of any control is visualized in a way that allows the experimenter to pass or fail a run. Second, the “Minimum Discriminatory Difference/Ratio” (MDD/MDR) is monitored as a measure of intra- and intersite assay variability, estimating whether a difference in the data generated for two compounds is likely to be a real difference or not.^6^ It does so by defining the statistically significant difference/ratio threshold at the 95% confidence level.

## Results

### Integration of Assay Workflows

The consideration of DMPK properties was historically achieved within our organization through ordering of bespoke assays by DMPK leads as motivated by project needs. The throughput of these assays varied considerably, ranging from tens of compounds per week to several hundreds, often necessitating prioritization between projects. To improve data coverage and strengthen our abilities to predict DMPK properties, we implemented the DMPK Wave 1 panel of assays.^6^ This panel provides weekly delivery of lipophilicity, solubility, PPB, and metabolic stability in rat hepatocytes and HLMs on all compounds synthesized globally within AstraZeneca. The integration into a combined panel includes a common ordering and delivery process and analytical method development that is shared between the five assays (*Fig. 1*).

Orders to the panel triggers a provisioning and shipping of samples (113 μL of a 10 mM stock solution in DMSO) when required to our R&D facility in Gothenburg. Service Level Agreement for these shipments is at 48 h for samples from the United Kingdom and 72 h for United States. Samples are collected during the week and an order for plating is submitted on Friday mornings, with deliveries before lunch of four sets of plates to support method development and the individual assays. These sets are complemented with plates holding compounds separately ordered to the respective assays. Analytical method development is initiated from one of the four sets of 96-well plates, with somewhere in the range of 150–300 compounds/week, using QuanOptimize (Waters) to generate MRM methods. This process runs over the weekend and involves ramping of collision energy and switching between positive and negative ionization modes.^9,10^ Established methods are saved in a MS/MS method library (see Materials and Methods section for details) for use with each assay.

This generic approach is successful in establishing methods for >95% of submitted compounds. Our process for failed compounds, for example, multicharged or in-source fragmented compounds, include assessment of identity and purity and subsequent full scans in an appropriate mass range (commonly 100–1,100 g/mol) to identify responses that can be traced back to the parent molecules. In a year, <3% of submitted compounds fail these routines and are investigated separately as motivated. Next all compounds are combined in pools of 2–10, ensuring maximal mass distribution, using in-house scripting routines (Microsoft Excel). These routines simultaneously generate run methods for all liquid handling and MS analysis steps. Finally, the utility of the established MRM methods is corroborated by means of chromatographic separation, investigating peak shape and area to generate UPLC-MS/MS methods. Finally, all samples are matrix matched and differ only in compound concentrations, except for any metabolites or other products formed.

Panel testing is achieved based on the three remaining plate sets (logD, Sol, and PPB share source plates) using a common liquid handling platform as detailed below for each assay. Peak identity and size are evaluated using TargetLynx (Waters) and subsequent evaluations of linearity in response and verifications of pool and run performance through QCs is achieved in Genedata Screener according to business rules. Importantly, retention time warnings are integrated into the evaluation templates, thus eliminating the risk that a peak from a potential metabolite, with the same molecular weight and MS fragment as a compound within the pool, is mistakenly integrated. Finally, qualified results are published in our database between Tuesday and Thursday of each week.

### Lipophilicity (logD7.4)

#### Background description

A critical physicochemical property that affects the availability of compounds in biological test systems, animal models of disease, and man is the lipophilicity. Described as one of the components of the Lipinski rule of five for drug-like molecules, this property reflects the distribution of compound between 1-octanol and an aqueous solution reported as a logP value (referred to as logD7.4 when the distribution extends to include also charged species).^11,12^ The original Lipinski rule pointed at the need for logP values below 5, while later refinements argues for a more narrow distribution with a desirable range for drug-like compounds between 1 and 3.^13^

Practically, these measurements can be achieved using one of two basic principles: (1) by sampling from octanol and buffer samples after thorough shaking and equilibration (shake-flask method)^14^ or (2) by a chromatographic method, in which the retention of compounds on a hydrophobic C18 column is measured and used as a proxy of the distribution by comparisons with standards with established logD7.4 values.^15,16^ The logD7.4 assay within our DMPK Wave 1 panel is conducted in a 96-deep well format at controlled pH (7.4) and represents a fully automated variant of the shake-flask methodology.^7^

#### Assay workflow

**Fig. 2.**
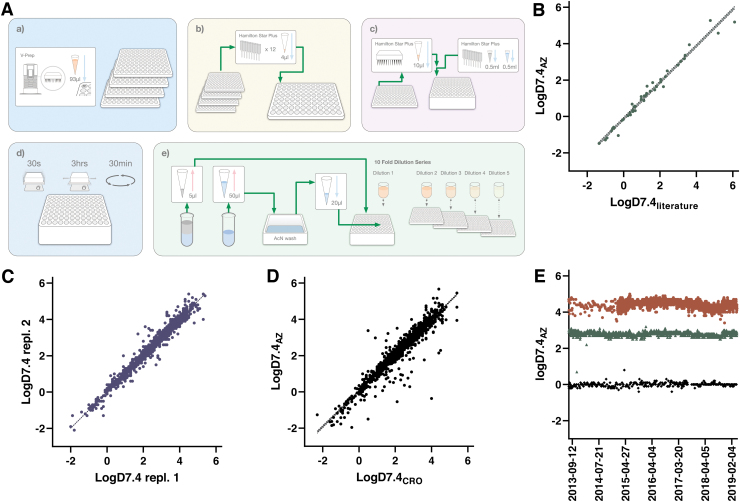
**(A)** Schematic illustration of the workflow for the logD7.4 assay. **(a**) Up to four compound plates with 93 μL in each well are obtained from Sample Management (the same source plates are used for the solubility and PPB assays). **(b)** Compound pools are created in an intermediate dilution plate through cherry-picking on a Hamilton STARplus. **(c)** Each pool is transferred to a deep well plate containing 1-octanol, mixed and complemented with aqueous buffer. **(d)** Samples are vortexed, equilibrated and centrifuged. **(e)** Sampling and serial dilution of both octanol and buffer phases is achieved on the same liquid handling station, with subsequent detection performed for the combined pools using UPLC-MS. **(B)** Correlation between data in the automated 96-well logD7.4 assay versus literature values for 44 commercially available compounds. The slope between average values from up to five independent measurements with previously reported data is 0.992 (*r*^2^ = 0.976). *Dotted lines* represent a 95% CI of 0.943–1.04. **(C)** Correlation between two independent experiments submitted from this assay to our internal database. When more than two independent replicates were available, we included the first two instances. The slope based on testing of 1,856 compounds comes out at 0.992 (*r*^2^ = 0.970) with a 95% CI of 0.984–1.00. **(D)** Correlation between harmonized assays at AstraZeneca Gothenburg (*y*-axis) and at Pharmaron (*x*-axis). The comparison is based on 1,682 compounds with a slope of 0.985 (*r*^2^ = 0.916) and a 95% CI of 0.970–0.999. **(E)** Manhattan plot following 6 years of operation from June 2013 to April 2019—nicardipine (*brown filled circle*), cyclobenzaprine (*green filled triangle*), and caffeine (*black filled diamond*). CI, confidence interval; PPB, plasma protein binding; UPLC, ultraperformance liquid chromatography. Color images are available online.

The logD7.4 assay workflow is schematically depicted in *Figure 2* and outlined in detail in *Table 1*. The assay starts with transfer of individual compound solutions into pools of 10 compounds, alongside two pool QCs, using a Hamilton STARplus liquid handling workstation. This pooling is achieved using unique run lists for each week, generated through in-house scripts based on delivery notes from Sample Management. This step combines 4 μL each of 10 mM compound solutions to a total of 48 μL in an intermediate plate (pool QCs are at 5 mM each). These and subsequent transfers as outlined below involve fresh pipetting tips to eliminate risks of cross-contamination.

**Table 1.** Protocol for the Shake-Flask logD7.4 Assay

Step	Parameter	Value	Description
1	Add octanol	500 μL	
2	Add compounds and controls	10 μL	10 test compounds (at 10 mM in DMSO) + two QCs (nicardipine and cyclobenzaprine at 5 mM each) are pooled
3	Add buffer	500 μL	Phosphate buffer pH 7.4
4	Vigorous shaking	1 min	
5	Equilibration shaking	3 h	Room temperature
6	Centrifugation	30 min, 3,000 × *g*	Room temperature
7	Octanol sampling	5 μL	
8	Removal of Octanol	495 μL	
9	Buffer sampling	20 μL	
10	Sample dilution series	1:10	Buffer samples are serially diluted (1:10) in acetonitrile/water (1:2, 0.1% FA, 2 nM verapamil—IS solution) in four steps and octanol samples in five steps
11	Plate sealing		
12	Assay readout	Peak area	LC/MS, TargetLynx
13	Data evaluation	logD7.4	Genedata Screener

**Step Notes**

1–3. Octanol, compounds and buffer are added to a 2 mL PP-based deep well plate (Product No.: 278752; Thermo Fisher Scientific) using a Hamilton STARplus. A PP-based microtiter plate (Product No.: 249944; Thermo Fisher Scientific) is used as an intermediate plate for the compound pooling.

4–5. The 2 mL plate is lidded (Product No.: 276002; Thermo Fisher Scientific) and shaken vigorously followed by equilibration under shaking for 3 h.

6. The phases are separated by centrifugation of the 2 mL plate in an Eppendorf 5810R (Eppendorf).

7–10. Sampling and removal of octanol is carried out on the Hamilton STARplus.

11. Plates are manually sealed with a 96-well cap natural seal (Product No.: 276002; Thermo Fisher Scientific).

12. The samples are analyzed by using a Waters iClass Acquity and Waters Xevo TQS. Chromatograms are evaluated using Waters TargetLynx software.

13. Data evaluation is achieved through Genedata Screener and approved results are reported to our internal database D360.

AcN, acetonitrile; DMSO, dimethyl sulfoxide; FA, formic acid; IS, internal standard; LC/MS, liquid chromatography/mass spectrometry; PP, polypropylene; QC, quality control.

The next assay step involves transfer of 500 μL octanol from a tray to an empty deep well plate, to which 10 μL of each pool is subsequently transferred and thoroughly mixed by repetitive aspiration and dispensing. An octanol-saturated phosphate buffer at pH 7.4 is then added (500 μL) followed by sealing of the content with a PP-based lid. Vigorous shaking of the content is achieved through vortexing for 30 s and equilibration of compound between the two phases is further established through a 3 h incubation on a plate shaker.

Following equilibration, the deep well plate is centrifuged to ensure separation between phases and the plate is next moved back to the Hamilton platform for sampling of 5 μL from the octanol phase. The sample is placed in a first dilution plate together with 495 μL of an AcN:H_2_O mix (1:2) containing FA (0.1%) and verapamil as internal standard (IS). The acidified and IS containing AcN solution, which is common for the logD7.4 and Sol assays, is referred to as the IS solution. The content is thoroughly mixed and next serially diluted to four additional plates with 10-fold dilutions to maximize the ability to detect weakly concentrated solutions and afford investigations of linearity in response.

After sampling, the remaining octanol is removed from the deep well plate through careful pipetting, followed by sampling from the aqueous buffer. This pipetting step is preceded by the prior sampling of a 5 μL plug of the AcN:H_2_O solution to avoid octanol contamination, and the pipette tips are additionally washed (see Assay validation and concordance testing section) before delivery of 20 μL samples from the aqueous solutions to the dilution plates. Also these samples are correspondingly diluted, and all plates are sealed with rubber lids that are compatible with the injection needles in the LC-MS instrumentation.

The plates are next brought to the MS instrumentation (Xevo TQS; Waters) and read out using the established UPLC-MS/MS methods. Run lists from the in-house scripts are imported into MassLynx to combine MRM methods and peak integration methods for all compounds within a pool using QuanOptimize. Data from each run are first processed in MassLynx and peak identities and integrated peak areas are next confirmed in TargetLynx. Confirmed data are exported to a raw data server for final evaluation in Genedata Screener. All aspects of the assays are documented in electronic laboratory notes, including compound identities, method descriptions, chromatograms, and a Genedata Screener report. Calculated logD values are exported into our internal database ensuring global availability to users.

#### Choice of validation test sets, QC compounds, and IS

A set of 44 commercially available compounds covering a logD7.4 range from −1.4 to 6.1 were selected for experimental validation of the automated method (*Supplementary Table S1*). We also included controls allowing for assessment of reproducibility between runs and between pools of compounds within each run (*Table 2*). These controls were chosen to reflect polar compounds with low logD7.4 values (caffeine at 0.1^17^), intermediate values (cyclobenzaprine at 2.8^8,18^), and more lipophilic compounds with high logD7.4 values (nicardipine at 4.5—in-house data).

**Table 2.** Quality Controls and Internal Standards

Name	Used in	Monoisotopic mass (g/mol)	MRM method	Cone Voltage (V)	Collision energy (V)
Caffeine	logD7.4	194.1	(+) 195.1–138.0	35	16
Cyclobenzaprine	logD7.4	275.2	(+) 276.0–216.0	35	22
Nicardipine	logD7.4	479.2	(+) 480.2–315.0	35	22
Verapamil	logD7.4, Sol, Mics, Heps	454.3	(+) 455.3–165.0	70	28
Astemizole	Sol	458.2	(+) 459.3–135.1	35	40
Phenytoin	Sol	252.1	(+) 253.1–182.1	35	16
Chlorpromazine	Sol	318.1	(+) 319.1–86.0	35	22
Warfarin	PPB	308.1	(+) 309.1–162.9	5	16
Propranolol	PPB	259.2	(+) 260.1–116.0	33	16
Metoprolol	PPB, Mics	267.2	(+) 268.2–116.3	35	16
5,5-diethyl-1,3-diphenyl-2-iminobarbituric acid	PPB, Mics, Heps	335.2	(+) 336.2–195.0	46	28
Diclofenac	Mics	295.0	(+) 296.0–214.2	35	40
Imipramine	Mics	280.2	(+) 281.4–86.1	35	16
Phenacetine	Mics	179.1	(+) 180.1–110.2	35	22
Benzydamine	Mics	309.2	(+) 310.2–86.1	35	16
Bosentan	Heps	551.2	(+) 552.2–202.0	35	28
Dofetilide	Heps	441.1	(+) 442.1–197.9	5	28
Indapamide	Heps	365.1	(+) 366.1–132.0	22	16
Lorazepam	Heps	320.0	(+) 321.0–274.9	35	22
Terfenadine	Heps	471.3	(+) 472.3–436.2	35	28

Heps, assay for metabolic stability in rat hepatocytes; Mics, assay for metabolic stability in human liver microsomes; PPB, plasma protein binding; Sol, solubility.

In our setup, caffeine is included randomly as a control between runs. Nicardipine is a good indicator of octanol contamination when sampling the buffer side and is therefore included in each pool and used to fail affected pools when the logD7.4 is lower than expected. Each pool also includes cyclobenzaprine with an intermediate logD7.4 to report on consistency between pools. To verify consistency between injection volumes, all samples contain 2 nM of verapamil (IS).

#### Assay validation and concordance testing

The key challenges associated with automation of the shake-flask assay concern (1) equilibration between phases in a microplate format; (2) octanol contamination of the aqueous phase when sampling; and (3) the large difference in concentrations between phases, which is especially pronounced for highly lipophilic compounds. The first challenge was solved through capping of the deep well plates and vigorous mixing of the two phases by vortexing followed by a 3 h equilibration period on a plate shaker. We compared the output of this approach with reference values taken from the literature^17^ as shown in *Figure 2B*, demonstrating that conditions closely resembling equilibrium was reached using this protocol and time period.

Automation of sampling from the aqueous phase presents significant challenges. Prior removal of the viscous octanol was achieved through a series of slow aspiration steps close to the buffer surface, with final aspiration achieved from within the aqueous solution. As already mentioned, contamination of any residual octanol, when sampling 50 μL of the buffer sample, is further prevented through the introduction of a plug in the tip before entering the aqueous phase.

We next discard 20 μL of this solution and wash the outside of the tips extensively by serial dipping into four independent AcN solutions. The sample is finally introduced into the dilution plate and the remainder of the solution discarded. The third challenge was addressed by automated 10-fold serial dilutions in multiple steps following sampling and assessment of linearity in response between dilutions, enabling measurements of logD values at either end of the scale from about −1 to 4. This was considered enough for a Wave 1 screening assay.

Establishment of the automated logD assay entailed testing of reproducibility as illustrated in *Figure 2C*, which compares data for all compounds that have been tested at a minimum of two different occasions in the assay (the first two instances were used for the illustration in case data are available for additional test occasions). Agreement between test occasions is excellent with few outliers (95% of compounds differing <0.3 between runs).

To ensure rapid availability of DMPK Wave 1 data for compounds synthesized at our external partner Pharmaron, efforts were put in place to fully harmonize this assay panel.^6^ A review of data in our database now includes a total of 898 compounds that have been tested both internally and at Pharmaron, with a comparison of results illustrated in *Figure 2D*. The data show acceptable agreement with 87% of compounds within 0.3 between sites. As described in detail below these assays have been running since 2013 and the performance of the QC compounds over a 6-year period at AstraZeneca R&D in Gothenburg is illustrated in *Figure 2E*. Except for a few occasions, the assay has been stably reporting expected values for these compounds with the largest variability observed for nicardipine, reflecting the challenges associated with octanol contamination when sampling the aqueous buffer.

### Solubility (Sol)

#### Background description

Solubility in aqueous solution is a parameter that determines whether a biological test system or organism is appropriately exposed to the compound. It also affects our ability to create suitable formulations for preclinical and clinical studies.^19,20^ For most test assays in early drug discovery, we report values based on nominal concentrations, that is, based on what is added to the test system, rather than performing actual measurements of these concentrations. This can be visible in concentration responses both in biochemical and cellular assay formats, where incomplete saturation or apparent bell-shaped responses can be observed for poorly soluble compounds simply because they fall out of solution.

Early solubility assessments are therefore critical for accurate interpretation of these responses and for understanding of structure-activity relationship (SAR). Solubility measurements can be achieved in several different ways and generally starts from known amounts of solid compound (low-throughput assays) or from DMSO stock solutions with or without removal of solvent.^21,22^ The solubility assay within the DMPK Wave 1 panel is based on the dried DMSO principle, is conducted in glass vials placed in a 96-vial rack holder, and represents a further miniaturized variant of previously published assays from AstraZeneca.^7,23^

#### Assay workflow

**Fig. 3.**
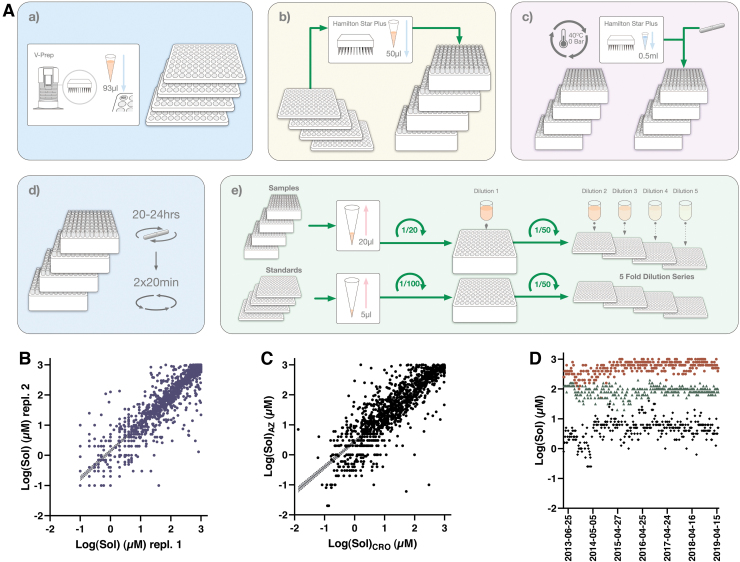
**(A)** Schematic illustration of the workflow for the Sol assay. **(a)** Samples are provided from the same source plates as those used for the logD7.4 and PPB assays. **(b)** Individual compound solutions are transferred to glass vials in 96-well racks. **(c)** DMSO is evaporated and dry compounds are solubilized in aqueous buffer and stirring bars are added to each vial. **(d)** Samples are equilibrated at 25°C with shaking for 20–24 h. Plates are centrifuged and cleared supernatants are transferred to fresh glass vials and centrifuged again. **(e)** Cleared supernatants are pooled (up to 10 samples/pool) and serially diluted in 50% acidified AcN:H_2_O with IS. Standard samples are treated similarly in a parallel workflow, with subsequent detection performed using UPLC-MS. **(B)** Correlation between two independent experiments submitted to the database. When more than two independent replicates were available, we included the first two instances. The slope based on testing of 1,219 compounds comes out at 0.911 (*r*^2^ = 0.814) with a 95% CI of 0.887–0.936. **(C)** Correlation between harmonized assays at AstraZeneca Gothenburg (*y*-axis) and at Pharmaron (*x*-axis). The comparison is based on 1,200 compounds with a slope of 0.847 (*r*^2^ = 0.749) and a 95% CI of 0.819–0.875. **(D)** Manhattan plot following 6 years of operation from June 2013 to April 2019—astemizole (*black filled diamond*), phenytoin (*green filled triangle*), and chlorpromazine (*brown filled circle*). AcN, acetonitrile; DMSO, dimethyl sulfoxide; IS, internal standard; Sol, solubility. Color images are available online.

The solubility workflow is illustrated in *Figure 3*, with a detailed description of each assay step in *Table 3*. Unlike the logD7.4 assay compounds are not pooled, but instead 50 μL of individual DMSO stock solutions are transferred to glass vials kept in a 3D printed rack that facilitates subsequent liquid transfers using a 96-well pipetting head on the Hamilton STARplus platform. There is no pool QC in the Sol workflow, instead this is replaced with the inclusion of three QCs, the positioning of which is randomized for each week. Solvent is evaporated and the dried compounds are resolubilized in 500 μL of aqueous phosphate buffer at pH 7.4.

**Table 3.** Protocol for the Dried Dimethyl Sulfoxide Solubility Assay

Step	Parameter	Value	Description
1	Transfer compounds to glass vials	50 μL	10 mM DMSO solution
2	Evaporate DMSO solvent	1 h	Evaporate solvent in a vacuum centrifuge at 40°C
3	Add buffer	500 μL	
4	Add stir sticks	1/well	
5	Equilibrate samples	20–24 h	Incubation at 25°C under stirring
6	Centrifugation	30 min, 2,000 × *g*	Room temperature, Eppendorf 5810R
7	Sample transfer	200 μL	Transfer cleared supernatants
8	Centrifugation	30 min, 2,000 × *g*	As in **6**
9	Sampling and pooling	20 μL	Sampling and pooling of 10 samples + twofold dilution of pool
10	Pooling of standards	5 μL	Pooling of 10 standards +10-fold dilution of pool
11	Establish dilution series of samples and standards	1:5	Serial dilution (1:5) in four steps
12	Plate sealing		
13	Assay readout	Peak area	LC/MS, TargetLynx
14	Data evaluation	μM	Genedata Screener

**Step Notes**

1. Compound stock solution is added to 1 mL Glass Flat Bottom Vials (Product No.: 4100FB-930VL; JG Finneran, ScanTec) situated in a 96-well rack using a Hamilton STARplus.

2. Solvent DMSO is evaporated in a Genevac HT-4 evaporator (Igenevac, Ipswich, Great Britain).

3. Phosphate buffer at pH 7.4 (*I* = 0.1 M) is added to the glass tubes containing dried compounds on a Hamilton STARplus.

4. Stir Stix from V&P Scientific (Product No.: VP 735-2).

5. Samples are incubated in an Eppendorf plate shaker (Eppendorf).

7. Supernatants are transferred to fresh glass tubes using a Hamilton STARplus.

9. Sampling and pooling of cleared buffer samples, including a twofold dilution in IS solution using a Hamilton STARplus.

10. Sampling and pooling of standards from DMSO stock solutions, including a tenfold dilution in IS solution using a Hamilton STARplus.

11. Samples and standards are further diluted 50-fold and then serially diluted in IS solution on a Hamilton STARplus.

12. Plates are manually sealed with a 96-well cap natural seal (Product No.: 276002; Thermo Fisher Scientific).

13. The samples are analyzed by using a Waters iClass Acquity and Waters Xevo TQS. Chromatograms are evaluated using Waters TargetLynx software.

14. Data evaluation is achieved through Genedata Screener and approved results are reported to our internal database D360.

Individual Stir Stixs are added to each glass vial using a drop dispenser from V&P Scientific, Inc., a rubber mat is used to seal the vials, and samples are then equilibrated under mixing for 20–24 h. The following day starts with removal of the Stir Stixs and subsequent centrifugation to pellet insoluble compound, before 200 μL of the supernatants are transferred to new glass vials on the Hamilton STARplus platform.

The process of centrifugation and transfer is repeated to remove any remaining debris, except that the 20 μL supernatant transfer combines sampling and pooling of 10 samples to a pool plate with 200 μL of an IS solution (same as in logD7.4). As mentioned previously, this pooling is achieved using unique run lists for each week. The liquid handling workflow is finished with transfer of 40 μL of the pooled samples to an analysis plate, where the samples are diluted fivefold in IS solution (160 μL). Standard curves are generated for each pool by combining and diluting 5 μL of each compound DMSO stock solution in the IS solution. The pooled samples are serially diluted in a four-point series to accommodate the span from 1 to 1,000 μM. Finally, sample and standard plates are sealed and read out, analyzed, and qualified as described for the logD7.4 assay. Calculated solubility values (in μM) are exported into our internal database ensuring global availability to users.

#### Choice of QC compounds and IS

While the performance of previous versions of our miniaturized dried DMSO solubility assay was validated through comparisons with a classical thermodynamic solubility test,^7,23^ in this study, we describe the inclusion of multiple QC compounds for assessment of assay performance between runs (*Table 2*). These were chosen to reflect the different solubility tiering and include astemizole at 2.9 μM, phenytoin at 86 μM, and chlorpromazine at 0.45 mM in published dried DMSO assays.^23^ These three compounds are included in each run with their glass vial positioning randomized to avoid any systematic bias. As for the logD7.4 assay, all samples also contain 2 nM of verapamil (IS) to verify consistency between injection volumes during analysis.

#### Assay validation and concordance testing

As described in the literature, there are several significant challenges associated with aqueous high-throughput solubility assays.^21,22^ Many of these are practical, such as achieving enough mixing in a glass vial format to establish equilibrium within a reasonable time frame (here 20–24 h), pelleting of insoluble compound and sampling of the clarified supernatant without contamination from nonpelleted microaggregates or flocculating compounds. Others are physicochemical in nature, for example, the potential formation of different crystalline forms with different dissolution rates in the dried DMSO assays, adding to variability in results between test occasions.

Results are also affected by the purity of each compound solution and this can differ depending on time of storage. In our semiautomated asthe practical challenges are mitigated by an overnight incubation under constant stirring to facilitate establishment of equilibrium as well as two cycles of centrifugation and aspiration to new containers to reduce contaminations from nonsoluble material. Even so, challenges remain for compounds that form microaggregates and flocculate and hence the automated assay, where visual inspection of individual samples is not included in our routines, produces valuable tiered Wave 1 results rather than precise measurements (*Fig. 3B*).

Illustrations of assay performance include reproducibility between separate test occasions as illustrated in *Figure 3B*, which compares data for all compounds that have been tested at least twice in the assay. Agreement between test occasions is considered reasonable (slope between occasions is at 0.91) given the significant challenges in miniaturized dried DMSO solubility assays, although it is immediately apparent from *Figure 3* that this method is associated with a larger variability than the other panel assays. A slightly weaker correlation (slope at 0.85) is observed for data generated in Gothenburg and at the CRO (*Fig. 3C*), which at the time of writing allowed comparisons across 1,200 compounds.

The larger variability for this assay is also apparent from the Manhattan plots monitoring performance of the QC compounds over a 6-year period (*Fig. 3D*), although at the same time it illustrates how solubilities can be tiered as low (<10 μM—availability in biological test assays can be severely limited), intermediate (<100 μM—likely available at screen concentration of 10 μM), and high (up to 1 mM—readily available in most test assays). Apart from the variability, which is especially pronounced for the poorly soluble astemizole, the assay has delivered stable results over this time period.

### Human PPB

#### Background description

PPB reflects the degree to which a compound is bound to proteins within blood, primarily to serum albumin (∼60%) and α1-acid glycoprotein, and to a lesser degree to lipoproteins and α, β‚ and γ globulins. Such binding directly impacts the exposure of relevant tissue as only unbound compound is available to interact with extracellular targets or partition over cell membranes to an intracellular site of action.^24,25^ Consequently, plasma proteins act as a reservoir from which bound compound is continuously released when unbound compound is taken up by cells, metabolized, or excreted. The parameter naturally affects also compound availability in cellular assays, which commonly include serum as a component of the cell medium.^26^

Measurements of PPB is therefore essential for accurate interpretation of cellular SAR, although there is still an on-going debate as to whether this parameter should be optimized.^27,28^ PPB measurements can be achieved using different principles, with equilibrium dialysis over a semipermeable membrane separating a plasma and a buffer-containing chamber as the most widely accepted method.^29–31^ Other approaches include ultrafiltration and ultracentrifugation methods as well as binding assays based on immobilized protein.^32^ The PPB assay within our panel is based on equilibrium dialysis and represents our implementation of a previously published method.^33^

#### Assay workflow

**Fig. 4.**
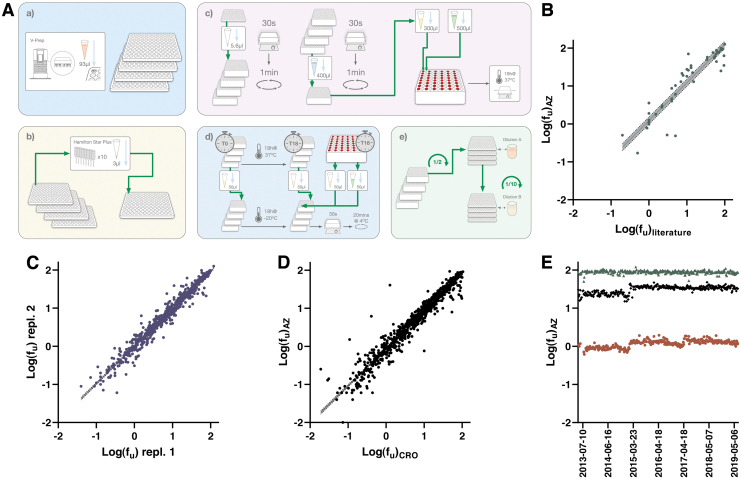
**(A)** Schematic illustration of the workflow for the PPB assay. **(a)** Samples are provided from the same source plates as those used for the logD7.4 and Sol assays. **(b)** Compounds are pooled in a pool plate on a Hamilton STARplus. **(c)** Compound pools are transferred to plasma-containing deep well plates (referred to as standard plates), mixed by shaking and centrifuged. Part of content is further diluted in an intermediate plate, while remaining content is serially diluted to create standard curves (*top*). Content in the intermediate plate is mixed by shaking and centrifuged before transfer of samples and buffer to the inward and outward chambers of Red Devices, respectively (*bottom*). **(d)** Time zero samples and standards are transferred from the intermediate plate to crash plates containing AcN with IS and matrix matched samples. The crash plate is kept in the freezer overnight. Incubated buffer and plasma samples from the Red Devices are transferred to the crash plate alongside plasma stability samples (T18) from the intermediate plate. The crash plate is thoroughly shaken and centrifuged cold. **(e)** Samples are diluted in acidified H_2_O and subsequently in acidified 37% AcN to and detected using UPLC-MS/MS. **(B)** Correlation between data in the automated PPB assay versus literature data for 47 drugs. The slope between average values from a minimum of three independent measurements with previously reported data is 0.964 (*r*^2^ = 0.834). *Dotted lines* represent the 95% CI 0.835–1.09. **(C)** Correlation between two independent experiments submitted to the database. When more than two independent replicates were available, we included the first two instances. The slope based on testing of 624 compounds comes out at 0.982 (*r*^2^ = 0.960) with a 95% CI of 0.966–0.997. **(D)** Correlation between harmonized assays at AstraZeneca Gothenburg (*y*-axis) and at Pharmaron (*x*-axis). The comparison is based on 801 compounds with a slope of 0.977 (*r*^2^ = 0.938) and a 95% CI of 0.960–0.994. **(E)** Manhattan plot following 6 years of operation from May 2013 to June 2019—warfarin (*brown filled circle*), metoprolol (*green filled triangle*), and propranolol (*black filled diamond*). Color images are available online.

The semiautomated PPB assay workflow is schematically depicted in *Figure 4* and outlined in detail in *Table 4*. Compound pooling, including warfarin as QC in each pool, is achieved on the Hamilton liquid handling platform using unique run lists. Pooled compound solutions in DMSO are then diluted 140-fold to 7 μM in human plasma from BioIVT. This plasma is recovered from whole blood donations (50 donors, equal number of males and females), pooled, aliquoted, and stored at −20°C until use.

**Table 4.** Protocol for the Human Plasma Protein Binding Assay

Step	Parameter	Value	Description
1	Thaw human plasma	3 min, 750 rpm	Thawing and centrifugation at room temperature
2	Pooling compounds	3 μL	Nine test compounds + one QC (warfarin)
3	Dilute pools in plasma	5.6 μL	Add pooled compounds to 792 μL plasma (to 7 μM)
4	Sample pool aliquots and dilute to start concentration	400 μL	400 μL diluted pools +160 μL of predispensed plasma
5	Sampling (*t* = 0 h)	50 μL	Take *t* = 0 h control samples from the 5 μM deep well plate for the stability and recovery studies
6	Add samples to RED devices	300 μL	Place in RED device plasma chambers
7	Add phosphate buffer at pH 7.4	500 μL	Place in surrounding buffer chamber
8	Sealing		Mounting of breathable seal on RED Devices
9	Equilibrate samples	18 h, 500 rpm	Incubation with shaking at 37°C and 5% CO_2_
10	Generate standard curves		Serial dilution of 7 μM samples
11	Store in freezer	18 h, −20°C	Crash plates
12	Adjust samples to RT	15 min	RED devices, Stability & Recovery and crash plate
13	Centrifugation	1 min, 1,000 × *g*	Stability & Recovery and crash plate at RT, Eppendorf 5810R
14	Buffer sampling	50 μL	From RED devices buffer chambers to crash plates
15	Plasma sampling	50 μL	From RED devices plasma chambers to crash plates
16	Sampling (*t* = 18 h)	50 μL	From Stability & Recovery plate to crash plate
17	Lidding and vigorous shaking	30 s	Silicone Cap Mats
18	Centrifugation	20 min, 3,000 × *g*	4°C, Sigma 6–16K (SIGMA Laborzentrifugen GmbH)
19	Dilution and transfer	1:2 + 1:10	Crash plate content is diluted and transferred to analysis plates (H_2_O + 0.2% FA)
20	Plate sealing		
21	Assay readout	Peak area	LC/MS, TargetLynx
22	Data evaluation	*f_u_*, recovery, stability (%)	Genedata Screener

**Step Notes**

1–4. Compound pooling is achieved in a PP-based microtiter plate (Product No.: 249944; Thermo Fisher Scientific). Pools are mixed and then diluted in plasma predispensed to deep well plates (Product No.: 260252; Thermo Fisher Scientific). A portion of the diluted pools are further diluted to 5 μM in the same deep well plate type (herein referred to as the Stability & Recovery plate), while the remaining content is used for generation of standard curves (see further down in this Table footnote).

5. A *t* = 0 h control sample for the Stability & Recovery studies is sampled to a crash plate already containing 400 μL of cold IS solution.

6–9. Plasma samples and buffer are added to the RED devices, sealed (Product No.: BEM-1, Diversified Biotech) and incubated on an orbital shaker (Eppendorf) for 18 h in a Galaxy R incubator (Richmond Scientific) alongside the Stability & Recovery plate.

10. Samples (at 7 μM) from Step **2** are serially diluted in plasma to generate a standard curve from 1.4 nM to 7 μM on the Hamilton STARplus. 50 μL of these samples are immediately transferred to the crash plate (see Step **5**).

11. Crash plates are stored at −20°C overnight.

12. The Stability & Recovery plate is vortexed before the centrifugation step.

14–16. Sampling is carried out manually with an 8-channel pipette to crash plates already containing 400 μL of cold IS solution and either 50 μL buffer or plasma to ensure matrix matching between samples.

17. Crash plates are lidded using silicone cap mats (Product No.: 276002; Thermo Fisher Scientific) and vortexed.

19. Crash plate content is diluted in two steps, starting with transfer and mixing of 75–75 μL of acidified water in a PP-based microtiter plate (Product No.: 249944; Thermo Fisher Scientific) on the Hamilton STARplus liquid handler. This content is further diluted 20–180 μL of acidified AcN:H_2_O (1:3, 0.1% FA).

20. Plates are manually sealed with a 96-well cap natural seal (Product No.: 276002; Thermo Fisher Scientific).

21. The samples are analyzed by using a Waters iClass Acquity and Waters Xevo TQS. Chromatograms are evaluated using Waters TargetLynx software.

22. Data evaluation is achieved through Genedata Screener and approved results are reported to our internal database D360.

PP, polypropylene.

This intermediate solution is used for two purposes: (1) to establish standard curves in plasma; and (2) for further dilution to 5 μM before addition of samples to the inner chamber of the rapid equilibrium dialysis (RED) devices. A phosphate buffer at pH 7.4 is added to the surrounding chamber to allow equilibrium dialysis over a semipermeable membrane with a molecular weight cutoff at 8 kDa. The filled RED devices are incubated on an orbital shaker for 18 h. Besides addition to the RED devices, the 5 μM solutions are also used to establish compound stability in plasma and recovery (mass balance) in the RED devices, with sampling performed at both the 0 and 18 h timepoints.

While the 18 h samples are incubated alongside the RED Devices, the 0 h samples are immediately quenched through addition to cold acidified AcN, containing 10 nM 5,5-Diethyl-1,3-diphenyl-2-iminobarbituric acid as IS, to precipitate proteins and prevent degradation. To ensure matching of sample matrices in these crash plates, a corresponding volume of phosphate buffer—in case of sampling plasma—or plasma—in case of sampling buffer—is added alongside AcN. A seven-point calibration curve in plasma, ranging from 1.4 nM to 7 μM, is also prepared for each pool through serial dilution of the 7 μM solution on the Hamilton liquid handling platform. These samples are also transferred to the crash plates and kept at −20°C until further processed for analysis the following day.

Following equilibration, all samples are brought to room temperature before sampling. This process includes centrifugation of the thawed crash plates before lids are removed. Sampling of 50 μL from the RED devices and the Stability & Recovery plate into crash plates is handled through manual pipetting, followed by lidding and vortexing of the crash plates to ensure adequate mixing. The assay procedure is finalized with a centrifugation step and transfer and twofold dilution of 75 μL of the supernatants in acidified H_2_O (0.2% FA) in a first set of high concentration analysis plates. A further 10-fold dilution is performed in acidified AcN:H_2_O (1:3; 0.1% FA) in a low concentration set of analysis plates, which are used primarily for the analysis to avoid signal saturation (the high concentration plates remain as backups should the signals be inadequate).

Analysis plates are finally sealed and read out, analyzed, and qualified as described above to enable export of calculated *f_u_*, stability, and recovery values (all in %) to our internal database. If the recovery deviates significantly from 100%, it indicates binding to the dialysis equipment or solubility issues. If the compound is unstable in plasma under the described assay conditions, it complicates interpretation of the assay data. The *f_u_* values are therefore only reported as aggregated data when both recovery and stability are >50%.

#### Choice of validation test sets, QC compounds, and IS

While a set of 72 internal compounds covering a *f_u_* range from 0.06% to 100% were originally used for experimental validation of the herein described automated method, here we examined our internal database for experimental values of approved drugs with well-established literature values and identified 50 drugs^31,33–35^ (detailed in *Supplementary Table S1*). As with the other panel assays, we include multiple controls allowing for assessment of reproducibility between runs and between pools of compounds within each run (*Table 2*). While propranolol reflects compounds with an intermediate *f_u_* in the 10%–30% range,^31,36^ metoprolol validates assay performance for compounds with limited PPB (*f_u_* ∼85%).^36^

During the validation work, it was observed in some pools that extensively bound compounds showed higher fraction unbound than expected. Closer inspection of these pools suggested that the reason was leakage of plasma proteins into the buffer side as previously reported.^37^ To control for potential leakage of the RED Device insert membrane, warfarin with an *f_u_* of ∼1%^33^ is included in each pool and used to fail affected pools when the measured *f_u_* is >2%. To achieve this, it is included in each pool and used to fail affected pools when the measured *f_u_* is >2%. All samples also contain 50 nM of 5,5-diethyl-1,3-diphenyl-2-iminobarbituric acid (IS) to allow for normalization between small variations in injection volume in the UPLC-MS/MS instrumentation.

#### Assay validation and concordance testing

The herein described PPB assay represents a further development of an in-house protocol^31^ as implemented with commercially available RED devices dialysis chambers.^33^ The weekly flow-through of compounds is accommodated at an acceptable cost by pooling,^31^ such that 10 compounds are dialyzed in parallel in each RED device. While the miniaturized assay does carry a risk of nonspecific binding to semipermeable membranes and plastic surfaces in the dialysis chambers, it is carefully controlled in our setup through parallel measurements of compound stability and recovery. There is also a potential risk of interference between compounds in their competition for available binding sites to plasma proteins.

To put this in perspective, each compound is available at 5 μM concentration (50 μM total compound in each pool), while the availability of binding sites on albumin is >500 μM.^38^ For human alpha-1-acid glycoprotein, the estimated plasma concentration is around 15–30 μM,^39^ such that the risk of interference is larger should more than one compound in the pool bind extensively to this protein. As illustrated below, the agreement between data from different test occasions and between test sites, which in practice translates to different pools, suggest that this problem is limited but remains a consideration when interpreting data. The assay also reports back on the 18 h plasma stability of each compound through parallel incubations. Assay performance as well as regular testing and acceptance of new batches of human plasma is achieved through a validation set, the composition of which has been continuously updated to reflect the chemical space of on-going drug discovery projects.

A comparison with literature data is shown in *Figure 4B*, demonstrating excellent agreement, especially considering the known variability between batches of plasma from different donors. Internal assay performance is further illustrated by a comparison of data from different test occasions in *Figure 4C*, where a slope close to unity confirms agreement between data from independent test runs. The strong correlation between data holds true when comparing data from the harmonized panels at AstraZeneca and the CRO (*Fig. 4D*), for which changes in plasma batches are synchronized. As shown in the Manhattan plot in *Figure 4E*, the assay has reported stable values for the QCs over a 6-year interval.

### Human Liver Microsomal Stability and Rat Hepatocyte Stability

#### Background description

Exposure of compounds in animal models of disease and man is dependent on uptake through the chosen route of administration, distribution, metabolism, and excretion, with metabolism reflecting the liability (tendency) of the drug to biochemical transformation.^40^ Metabolic stability is therefore an essential parameter in compound optimization,^41,42^ and it can be studied using suitable *in vitro* model systems. Most commonly, these are based on metabolically active hepatocytes or isolated liver microsomes,^43^ both of which can be obtained from different species. Comparisons across these model systems are required for prediction of exposure and reliable interpretation of data from *in vivo* studies in animal models of disease.

Comparison of stabilities in these two types of model systems can also help differentiate between phase I and phase II biotransformation.^43^ While phase I transformation (microsomes and hepatocytes) modifies molecules by introducing functional groups through oxidation, reduction, and hydrolysis, thus making compounds more polar and easily excreted, phase II transformations (hepatocytes) incorporate endogenous molecules such as glucuronic acid, sulfuric acid, and acetic acid through conjugation. It is important to consider that stability measurements are influenced by compound availability in the test system, thus requiring suitable measurements and compensations to accurately reflect intrinsic metabolic stability.^44^

Metabolic stability measurements are commonly conducted in a kinetic mode, where the degradation of molecules is followed over time after addition to the metabolically active system and there are several published variants of screen compatible formats.^45,46^ The Mics and Heps assay in our DMPK Wave 1 panel are fully automated assays conducted in 96-well format that represent further developments of previously published assays.^7^

#### Assay workflows

**Fig. 5.**
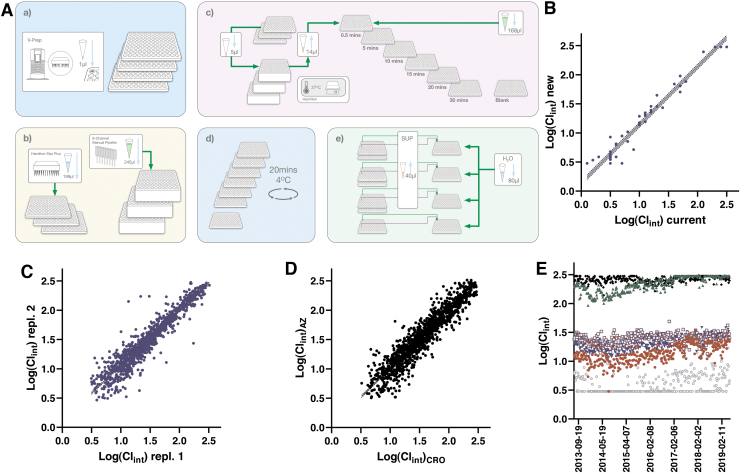
**(A)** Schematic illustration of the workflow for the Mics assay. **(a)** Up to three compound plates with 1 μL in each well are obtained from Sample Management. **(b)** The compounds are diluted in 50% AcN. Human liver microsomes diluted in NADPH containing PBS buffer are dispensed into a matching number of deep well plates. **(c)** The plates are heated to 37°C and incubations are subsequently started by addition of diluted compounds. Sampling, pooling and quenching is achieved at different time points on the Hamilton STARplus platform by adding aliquots to crash plates already holding AcN with IS. A blank plate with diluted liver microsomes is prepared separately. **(d)** Following incubations all plates are centrifuged to pellet the microsomes. **(e)** Final sampling of cleared supernatants is combined with additional pooling of plate content from the upper and lower parts of each plate into one half of a single analysis plate, that is, each analysis plate holds the content of two crash plates. These samples are diluted in predispensed H_2_O and subsequently detected using UPLC-MS/MS. **(B)** Illustration of recent data (2019) from a validation of a new batch of cryopreserved human liver microsomes. The slope based on testing of 46 compounds comes out at 0.980 (*r*^2^ = 0.951) with a 95% CI of 0.913–1.05. **(C)** Correlation between two independent experiments submitted to the database. When more than two independent replicates were available, we included the first two instances. The slope based on testing of 1,122 compounds comes out at 0.935 (*r*^2^ = 0.894) with a 95% CI of 0.916–0.954. **(D)** Correlation between harmonized assays at AstraZeneca Gothenburg (*y* axis) and at Pharmaron (*x* axis). The comparison is based on 1,313 compounds with a slope of 0.977 (*r*^2^ = 0.882) and a 95% CI of 0.957–0.996. **(E)** Manhattan plot following 6 years of operation from June 2013 to April 2019—diclofenac (*black filled diamond*), verapamil (*green filled triangle*), imipramine (*brown filled circle*), phenacetine (*purple unfilled square*), benzydamine (*blue filled triangle*), and metoprolol (*gray unfilled circle*). Mics, assay for metabolic stability in human liver microsomes. Color images are available online.

In line with previous descriptions, the workflow for the HLM assay is schematically depicted in *Figure 5* and further detailed in *Table 5*. The first assay step involves preparation of a homogeneous solution of HLMs, starting with thawing of commercial preparations from BioIVT at 37°C on a water bath and subsequent dilution in buffer with NADPH added as cofactor. This working solution is transferred to a set of up to three 96 deep well plates, which are placed on heated positions with shaking on the Hamilton STARplus liquid handler.

**Table 5.** Protocol for the Human Liver Microsome Stability Assay

Step	Parameter	Value	Description
1	Add liver microsomes	245 μL	Prepare and add working solution
2	Preincubation	≤3	37°C and continuous shaking
3	Dilute compounds and controls	1 μL	Test compounds + six QCs (diclofenac, imipramine, metoprolol, benzydamine, phenacetin and verapamil)
4	Start reactions	5 μL	
5	Continuous sampling, pooling and quenching	20 μL	Aliquots taken at 0.5, 5, 10, 15, 20, and 30 min
6	Centrifugation	20 min, 3,000 × *g*	4°C, Sigma 6–16K (SIGMA Laborzentrifugen GmbH)
7	Sampling and dilution	2 × 40 μL	Supernatants are further pooled and diluted with deionized water
8	Plate sealing		
9	Assay readout	Peak area	LC/MS, TargetLynx
10	Data evaluation	Cl_int_	Genedata Screener

**Step Notes**

1. HLMs are thawed at 37°C and diluted at room temperature with phosphate buffer at pH 7.4 alongside freshly prepared NADPH to reach a concentration of 1 mg protein/mL and 1 mM NADPH. 245 μL is distributed to wells of up to three 1 mL PP deep well plates (Product No.: 278752; Thermo Fisher Scientific). Plates are placed on a shaker and heating position on the Hamilton STARplus platform (kept at 37°C while shaking at 800 rpm).

2. A maximal number of three plates are placed on a Hamilton STARplus platform and preincubated under continuous shaking until all wells are at 37°C (∼15 min).

3. Compound and control stock solutions (1 μL at 10 mM) are diluted to 50 μM by adding 199 μL of AcN:H_2_O (1:2) using a Hamilton STARplus. The six QCs are used to monitor specific human P450 isoform activities: diclofenac (2C9), imipramine (2C19), metoprolol (2D6), benzydamine (flavin-containing monooxygenases), phenacetin (1A2), and verapamil (3A4).

4. Reactions are started by transfer of a 5 μL aliquot from the 50 μM solutions to preheated microsomes at 37°C on the Hamilton STARplus platform. The diluted microsomes remain on the shaker throughout the experiment.

5. Samples are aliquoted from reaction plates 1–3 at different time points and pooled (1–3 compounds depending on number of reaction plates) in cooled AcN with 20 nM 5,5-Diethyl-1,3-diphenyl-2-iminobarbituric acid to quench the reactions in a PP-based microtiter plate (Product No.: 249944; Thermo Fisher Scientific). The AcN volume is matched with the number of incubation plates, that is, from 80 μL (1 plate) to 240 μL (3 plates). This step generates one quench plate per time point, all of which are lidded with a microplate lid (Product No.: 263339; Thermo Fisher Scientific).

7. The content in wells A1 through to D12 are pooled with the content in wells E1 through to H12 (top half with bottom half) for each quench plate (time point) and diluted twofold with water (80 μL in each well is first placed in each well) in PP-based microtiter plates (Product No.: 249944; Thermo Fisher Scientific).

8. Plates are manually sealed with a 96 well cap natural seal (Product No.: 276002; Thermo Fisher Scientific).

9. The samples are analyzed by using a Waters iClass Acquity and Waters Xevo TQS. Chromatograms are evaluated using Waters TargetLynx software.

10. Data evaluation is achieved through Genedata Screener and approved results are reported to our internal database D360.

HLM, human liver microsome.

The next step involves dilution of test compounds and QCs to 50 μM, while the HLM working solutions are preincubated to reach 37°C. Reactions are started by adding the diluted compounds to the preincubations, and following mixing, by repetitive aspiration and dispensing, sampling is continuously achieved from the same reaction mixtures under a 30 min time period. Sampled volumes are immediately quenched in predispensed IS solution in “quench plates,” where the contents from up to three reaction plates are pooled. This procedure contrasts from other published automated HLM assays, where separate reaction plates are used for each time point, with the procedure as described herein allowing testing of up to 282 compounds (+6 QCs) across three deep well plates and seven quench plates.

Precipitated proteins in the IS solution are pelleted through centrifugation and clarified supernatants are further pooled by a factor of two by combining the upper and lower content of the 96-well plates in analysis plates (*Fig. 5A*). These are sealed and read out as described for the other assays, with quantification of peak areas in TargetLynx and further analysis using dedicated templates within Genedata Screener to calculate intrinsic clearance rates (Cl_int_).

**Fig. 6.**
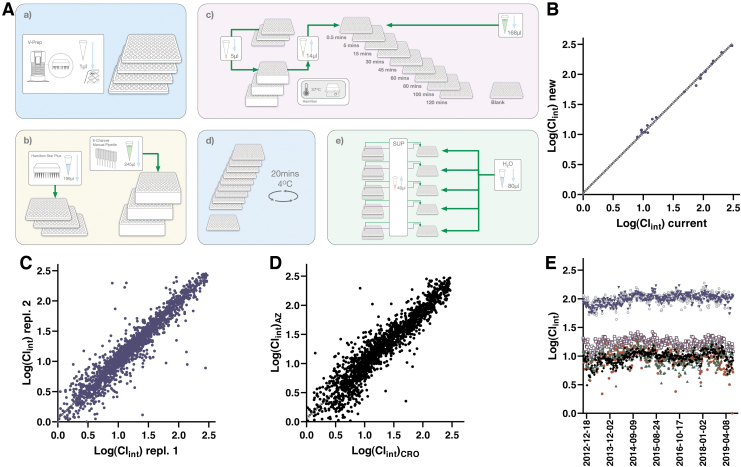
**(A)** Schematic illustration of the workflow for the Heps assay. **(a)** Up to three compound plates with 1 μL in each well are obtained from Sample Management. **(b)** The compounds are diluted in 50% AcN. A homogeneous suspension of rat hepatocytes is dispensed into a matching number of deep well plates. **(c)** The incubations follow the same procedure as the Mics assay except for the exact timings and choice of QCs. **(d)** Following incubations all plates are centrifuged to pellet the hepatocytes. **(e)** Sampling, pooling, dilution and detection is performed as described for the Mics assay. **(B)** Illustration of recent data (2019) from a validation of a new batch of cryopreserved rat hepatocytes. The slope based on testing of 24 compounds comes out at 0.985 (*r*^2^ = 0.996) with a 95% CI of 0.958–1.01. **(C)** Correlation between two independent experiments submitted to the database. When more than two independent replicates were available, we included the first two instances. The slope based on testing of 1,825 compounds comes out at 0.954 (*r*^2^ = 0.906) with a 95% CI of 0.940–0.968. **(D)** Correlation between harmonized assays at AstraZeneca Gothenburg (*y* axis) and at Pharmaron (*x* axis). The comparison is based on 1,679 compounds with a slope of 0.929 (*r*^2^ = 0.881) and a 95% CI of 0.913–0.946. **(E)** Manhattan plot following 6 years of operation from October 2012 to April 2019—bosentan (*black filled diamond*), dofetilide (*green filled triangle*), indapamide (*brown filled circle*), lorazepam (*purple unfilled square*), terfenadine (*blue filled triangle*), and verapamil (*gray unfilled circle*). Heps, assay for metabolic stability in rat hepatocytes; QC, quality control. Color images are available online.

The assay procedure for the rat hepatocyte stability assay closely resembles that of the Mics assay as described in detail in *Figure 6A* and *Table 6*. Small differences between these assays remain for historical reasons, but the main essential differences are the introduction of cryopreserved rat hepatocytes instead of the HLMs and the use of a different set of QCs and validation sets to reflect the different metabolism. The sampling from the same deep well plate over a 2 h time period puts significant demands on continuous mixing to retain the hepatocytes in suspension, which is achieved through plate shaker positions on the Hamilton STARplus station.

**Table 6.** Protocol for the Rat Hepatocyte Stability Assay

Step	Parameter	Value	Description
1	Add rat hepatocyte suspension	245 μL	10^6^ cells/mL and viability >80%
2	Preincubation	≤3	37°C and continuous shaking
3	Dilute compounds and controls	1 μL	Test compounds + six QCs (bosentan, dofetilide, indapamide, terfenadine, verapamil, and lorazepam)
4	Start reactions	5 μL	
5	Continuous sampling, pooling and quenching	15 μL	Aliquots taken at 0.5, 5, 15, 30, 45, 60, 80, 100, and 120 min
6	Centrifugation	20 min, 3,000 × *g*	4°C, Sigma 6–16K (SIGMA Laborzentrifugen GmbH)
7	Sampling and dilution	2 × 40 μL	Supernatants are further pooled and diluted with deionized water
8	Plate sealing		
9	Assay readout	Peak area	LC/MS, TargetLynx
10	Data evaluation	Cl_int_	Genedata Screener

**Step Notes**

1. Cryopreserved male Wistar han rat hepatocytes are rapidly thawed in a water bath at 37°C and washed in 50 mL prewarmed (37°C) Leibovitz medium pH 7.4.^58^ The cell suspension is centrifuged for 3 min at 50 rpm, supernatant is removed, and pellet is resuspended (repeated twice). After automated cell counting the suspension is diluted to a final concentration of 10^6^ cells/mL in Leibovitz medium and 245 μL of the cell suspension is manually transferred to 1 mL PP deep well plates (Product No.: 278752; Thermo Fisher Scientific) using a multipipette.

2. A maximal number of three plates are placed on a Hamilton STARplus platform and preincubated under continuous shaking until all wells are at 37°C (∼15 min).

3. Compound and control stock solutions (1 μL at 10 mM) are diluted to 50 μM by adding 199 μL of AcN:H_2_O (1:2) using a Hamilton STARplus. Five of the QCs were inherited from previous protocols and reflect important CYP 3A4 metabolism in man: bosentan, dofetilide, indapamide, terfenadine, and verapamil, while lorazepam controls for phase II metabolism (UGT2B15).

4. Reactions are started by transfer of a 5 μL aliquot from the 50 μM solutions to preheated hepatocyte suspensions. The reaction mixtures remain on the shaker throughout the experiment.

5. Samples are aliquoted from reaction plates 1–3 at different time points and pooled (1–3 compounds depending on number of reaction plates) in cooled acidified AcN (0.1% FA) with 20 nM 5,5-Diethyl-1,3-diphenyl-2-iminobarbituric acid to quench the reactions in a PP-based microtiter plate (Product No.: 249944; Thermo Fisher Scientific). The AcN volume is matched with the number of incubation plates, that is, from 60 μL (1 plate) to 180 μL (3 plates). This step generates one quench plate per time point, all of which are lidded with a microplate lid (Product No.: 263339; Thermo Fisher Scientific).

7. The content in wells A1 through to D12 are pooled with the content in wells E1 through to H12 (top half with bottom half) for each quench plate (time point) and diluted twofold with water (80 μL in each well is first placed in each well) in PP-based microtiter plates (Product No.: 249944; Thermo Fisher Scientific).

8. Plates are manually sealed with a 96 well cap natural seal (Product No.: 276002; Thermo Fisher Scientific).

9. The samples are analyzed by using a Waters iClass Acquity and Waters Xevo TQS. Chromatograms are evaluated using Waters TargetLynx software.

10. Data evaluation is achieved through Genedata Screener and approved results are reported to our internal database D360.

#### Choice of validation test sets, QC compounds, and IS

Compound validation sets, representing a range of different structural classes and metabolic stabilities (1 ≤ Cl_int_ ≤ 300), are in use for regular introduction of new batches of HLM and cryopreserved hepatocytes. The compositions of these test sets are detailed in *Supplementary Table S1*. Agreement is required for adoption of new batches, with the cutoff set at a maximal twofold average difference across the compounds in the validation sets. About one in four new batches of rat hepatocytes fail to comply with this criterion, such that it is returned to the vendor, whereas the failure rate is smaller for the HLM batches.

Multiple controls are included in the assays to reflect metabolism through different human P450 isoforms in the Mics QC set and with focus on CYP 3A4 metabolism and phase II elimination in the Heps set (detailed in *Table 2*). These were also chosen to reflect fast, intermediate, and slow metabolism such that the test runs can be failed when results for the QCs fall outside of norm. As for the PPB assay, all samples contain 20 nM of 5,5-diethyl-1,3-diphenyl-2-iminobarbituric acid as IS to allow for normalization of injection volumes.

#### Assay validation and concordance testing

While absolute comparisons with published metabolic stabilities are challenging given their dependence on the input material, especially in the case of the primary rat hepatocytes as this depends on strains and gender, it is important that the test results allow comparisons over an extended time interval. This can only be achieved through careful selection and control of input material, that is, through comparisons of assay data between different batches of HLM and rat hepatocytes. To illustrate such assay concordance, we include the results from the last batch validations in 2019, which are provided in *Figures 5B* and *6B*, respectively, where data obtained with the new reagents are compared with current production batches. Excellent agreement is observed in both cases with slopes close to unity and with much smaller deviation from the accepted average two-fold differences.

Further illustrations of assay performance over the 6-year period of operation can be seen by the comparison of test results from two independent test occasions as illustrated in *Figures 5C* and *6C* for the Mics and Heps assays, respectively, and correspondingly between sites in *Figures 5D and 6D*. Performance over the full-time period can also be evaluated through the Manhattan plots in *Figures 5E and 6E*, both of which demonstrate excellent assay stability over time. Such performance has allowed our organization to build reliable prediction models and to compare results over time.

#### Assay panel metrics and examples of project impact

**Fig. 7.**
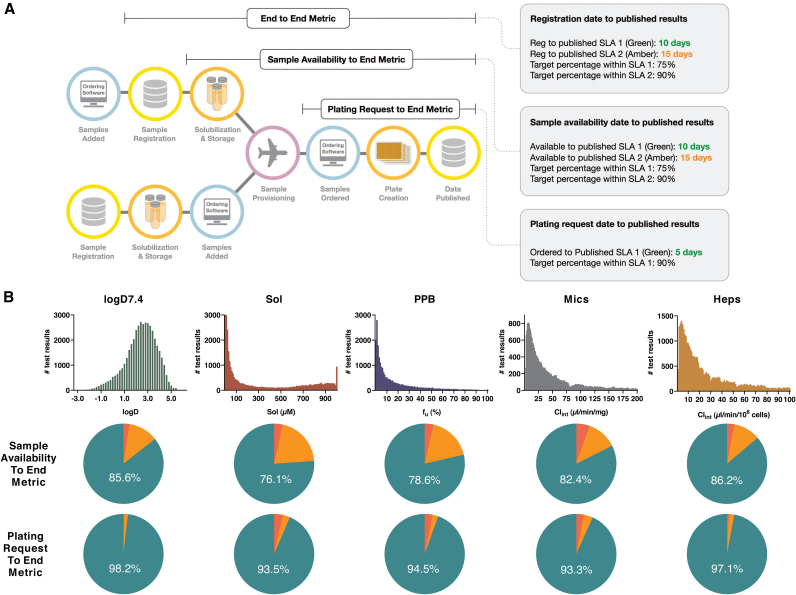
**(A)** Schematic illustration of internal metrics designed to measure times from registration of samples to the appearance of experimental data in internal databases (SLA). Measurements include the full “End to End Metric”—starting with sample registration, “Sample Availability to End Metric”—starting from when a physical sample becomes available at a global sample management facility (probing shipping efficiency and assay performance) and “Plating Request to End Metric”—from ordering of locally available compounds to publication of assay results (probing assay performance). **(B)** *Top*—Histograms of panel results since the start of operations in 2013 through to May 2019. *Mid*–pie charts illustrating number of days from samples being physically available at a Sample Management site to published data in database (combined statistics from 2018 and 2019). *Green* <10 days, *orange* <15 days and *red* if outside of SLA. *Bottom*—corresponding pie charts for the number of days from plating request to published data. *Green* <5 days, *orange* <10 days and *red* if outside of SLA. SLA, Service Level Agreement. Color images are available online.

Delivery of timely and harmonized data across a global R&D organization requires optimization of all steps, from registration and shipping of physical samples to operation of the assay panel itself. Measures are in place to probe the performance of each of these steps (*Fig. 7A*), that is, from original registration of new samples at any site, their physical arrival at each local sample management facility, subsequent shipping and delivery, local sample solubilization and plating to assays, and finally operations of all assays through to publication of quality-controlled data. Here, we focus this analysis to the latter steps, which probes timings from physical availability at any global sample management facility and from when compounds are ordered to the panel test, respectively, as these are important metrics for internal DMTA testing.

Following 6 years of operation, there is now a total of 229,354 data points reported from the assay panel (not including data from the harmonized panel at Pharmaron), approximately evenly spread between the five assays (data retrieved on May 14, 2019). Historical data are presented in the form of histograms in *Figure 7B*, while an example of how these properties are optimized throughout a project lifetime is provided below. Below each histogram are pie charts where samples that meet timelines are given as green if qualified results are published within 5 days from ordering of compounds to test and 10 days from physical sample availability at a sample management facility, orange if within 10 or 15 days, respectively, and red if outside of these timelines. Given excellent historical performance of internal shipping and the DMPK Wave 1 panel, our current focus is toward further improving the flow of samples from internal and external chemistry organizations to Sample Management.

**Fig. 8.**
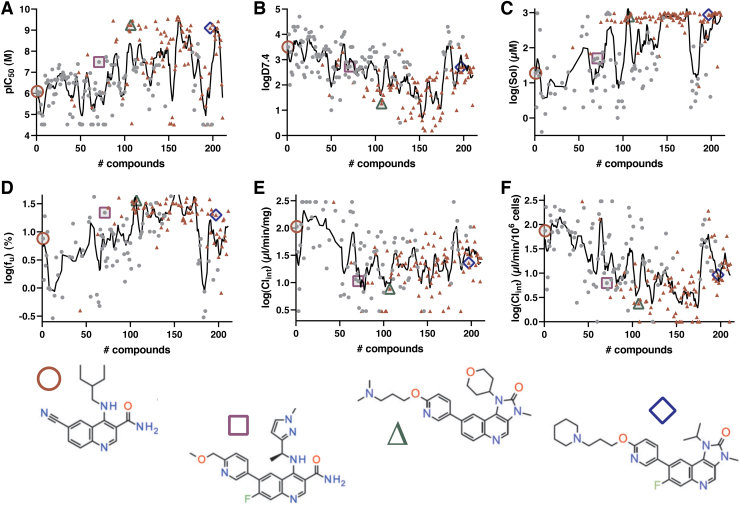
**(A)** Illustration of data obtained in the ATM inhibitor project, separated on neutral compounds (*gray filled circle*) and bases (*brown filled triangle*). **(A)** Potency on primary target reported as the negative logarithm of apparent IC_50_ values. **(B)** logD7.4. **(C)** Logarithm of the aqueous solubility (μM). **(D)** Logarithm of the fraction unbound (*f_u_*) obtained in the PPB assay. **(E)** Logarithm of the intrinsic clearance values in human liver microsomes. **(F)** Logarithm of the intrinsic clearance values in primary rat hepatocytes. The *lines* in each graph represent the moving average for the compounds (each period defined by 10 compounds). The chemical structures of the published chemical starting point (*brown unfilled circle*), an optimized lead compound (*purple unfilled square*) and two candidate drugs (*green unfilled triangle*, *blue unfilled diamond*) are provided below the graphs. ATM, Ataxia Telangiectasia Mutated; PPB, plasma protein binding. Color images are available online.

The value of the panel data for the optimization of internal prediction models has already been described,^47^ tools that are continuously applied for characterization of virtual compound sets and associated decision-making for compound synthesis. Here, we additionally illustrate the role of panel assay results in the optimization of inhibitors of the Ataxia Telangiectasia Mutated (ATM) kinase (*Fig. 8*). While the initial starting point showed μM potency on the target protein with a high logD7.4 value of 3.5, low μM solubility, and high intrinsic clearance, a published intermediate compound had improved considerably on all these aspects.^48^ As illustrated in *Figure 8*, further optimization yielded a published candidate drug^49^ with sub-nM potency and significant improvements in all DMPK Wave 1 measures, especially with regard to aqueous solubility that exceeded the assay limit of 1 mM.

What is noticeable from these comparisons is that later optimization efforts toward a candidate drug with improved availability in CNS showed a return in the logD value to 2.7, while retaining solubility, metabolic stability, and potency for the target.^50,51^ Collectively, these data demonstrate how the availability of high-throughput quality DMPK assays for every round in the DMTA cycle allows identification of leads with excellent properties that can be progressed to candidate drugs.

## Discussion

The DMTA cycle is central to drug discovery, serving to generate hypotheses that can be experimentally tested through iterative compound synthesis and testing.^52^ While progress on the biology of interest is monitored through biochemical and cellular SAR profiling assays, such optimization also includes physicochemical and metabolic properties that affect availability in biological test systems and in man. Given the importance of early consideration of these aspects, AstraZeneca took a strategic decision in 2012 to implement a DMPK Wave 1 panel of assays, with the intent to harmonize and broaden the delivery of relevant, high-quality early DMPK data.

Each of the panel assays were carefully selected to provide a fingerprint that enables ranking of compounds and identification of liabilities within a compound series. Assays for lipophilicity, solubility, human PPB, and metabolic stability in both rat hepatocytes and HLMs were included, as data from these assays constitute the basis for estimating human pharmacokinetic parameters and oral bioavailability, and in combination with target potency can provide early human dose predictions (eD2M).^53^

Lipophilicity is described as a partition coefficient, logD7.4, which is defined as the ratio of compound concentration at equilibrium between 1-octanol and an aqueous phosphate buffer at pH7.4. Lipophilicity correlates with many other properties and is the main descriptor for prediction of several parameters related to metabolism, absorption, distribution, and potency.^13^ One of these is the aqueous solubility, a property that influences the oral bioavailability of candidate drugs. It is challenging to develop formulations for poorly soluble compounds,^54,55^ such that early identification of solubility limitations in a compound series enables design of compounds with greater likelihood for good bioavailability in clinical settings. Another parameter that affects drug disposition is the PPB, which is used together with metabolic stability data to predict hepatic clearance. Although human PPB is an important property to measure, it is generally not a property that should be optimized.^24,26–28^

Various methods investigating *in vitro* metabolic stability are applied in drug discovery to predict the *in vivo* metabolism of compounds.^42,56^ The most commonly used approaches are incubations in HLMs, which mainly contain enzymes responsible for oxidative metabolism such as cytochrome P450s (CYPs).^57^ Since most marketed drugs are predominantly cleared by hepatic CYP-mediated metabolism, HLMs are ideal for studying *in vitro* intrinsic clearance and to predict human clearance. Metabolic stability in rat hepatocytes is primarily screened to additionally inform about the ability to predict the *in vivo* pharmacokinetics from *in vitro* data for a compound series.

A good *in vitro–in vivo* correlation in rats and other preclinical species will increase the confidence in the prediction of the human pharmacokinetic profile from *in vitro* data. In addition to oxidative metabolism that can be studied in microsomal incubations, measurements in rat hepatocytes include contributions of other enzymes and cofactors for conjugation to the metabolic processing of compounds.

In conclusion, this work describes an integrated and semiautomated workflow that takes experimental consideration of the above-mentioned properties. This five-assay DMPK Wave 1 panel ensures global availability and capacity to routinely accommodate all newly synthesized compounds within AstraZeneca and through CROs.^59^ As illustrated through detailed experimental descriptions, accompanied by illustrations of accumulated data following 6 years operations, the panel delivers quality data on physicochemical properties and metabolic stabilities concurrent with SAR-profiling assays, allowing for appropriate consideration of these aspects in a smooth and efficient DMTA process.

## Supplementary Material

Supplemental data

## Abbreviations Used

AcN: acetonitrile
ATM: Ataxia Telangiectasia mutated
CI: confidence interval
Cl_int_: intrinsic clearance rates
CRO: contract research organization
CYP: cytochrome P450
DMPK: drug metabolism and pharmacokinetic
DMSO: dimethyl sulfoxide
DMTA: design-make-test-analyze
FA: formic acid
Heps: assay for metabolic stability in rat hepatocytes
HLM: human liver microsomes
HPLC: high performance liquid chromatography
IS: internal standard
logD7.4: lipophilicity assay
Mics: assay for metabolic stability in human liver microsomes
MRM: multiple reactions monitoring
MS: mass spectrometry
PP: polypropylene
PPB: plasma protein binding
QC: quality control
RED: rapid equilibrium dialysis
SAR: structure-activity relationship
SLA: Service Level Agreement
Sol: solubility assay
UPLC: ultraperformance liquid chromatography
