# Supplementary material for: A Fully Integrated Assay Panel for Early Drug Metabolism and Pharmacokinetics Profiling
Source: Assay Drug Dev Technol. 2020 May 20;18(4):157–79. doi: 10.1089/adt.2020.970 (PMC7567642; doi:10.1089/adt.2020.970)
Supplement: Supplemental data [file Supp_Data.pdf]

Supplemental Data

A fully integrated assay panel for early DMPK profiling

Johan Wernevik[1,*], Fredrik Bergström[2], Anna Novén[1], Johan Hulthe[1], Linda Fredlund[1], Dan Addison[3], Jan Holmgren[4], Per-Erik Strömstedt[1], Erika Rehnström[5] and Thomas Lundbäck[1,*]

[1] Mechanistic Biology & Profiling, Discovery Sciences, R&D, AstraZeneca, Gothenburg, Sweden

[2] DMPK, Early CVRM, BioPharmaceuticals R&D, AstraZeneca, Gothenburg, Sweden

[3] Sample Management, Discovery Sciences, R&D, AstraZeneca, Cambridge, UK

[4] Sample Management, Discovery Sciences, R&D, AstraZeneca, Gothenburg, Sweden

[5] Clinical Sampling & Alliances, Precision Medicine, AstraZeneca, Gothenburg, Sweden

Running title: DMPK Wave 1 assay panel

* Corresponding authors:
johan.wernevik@astrazeneca.com
+46 (0)31-776 13 32
thomas.lundback@astrazeneca.com
+46 (0)72-711 66 89

Keywords: mass spectrometry, automation, DMPK, profiling, assay panels

Supplemental Table 1. Composition of validation sets

logD7.4	PPB	Mics	Heps
Acetaminophen	Acetaminophen	Acetaminophen	Bosentan
Alprenolol	Amiloride	Antipyrine	Caffeine
Amiodarone	Astemizole	Benzydamine	Carvedilol
Antipyrine	Atenolol	Bosentan	Diazepam
Atropine	Bepridil	Bufuralol	Diclofenac
Bifonazole	Bumetanide	Caffeine	Diflunisal
Caffeine	Chlorpromazine	Carvedilol	Dofetilide
Carbamazepine	Ciprofloxacin	Cefoperazone	Imipramine
Chloramphenicol	Clozapine	cerivastatin	Indapamide
Chlorpromazine	Desipramine	Chlordiazepoxide	Internal
Chlorthalidone	Diazepam	Desipramine	Internal
Cimetidine	Disopyramide	Diazepam	Internal
Clonidine	Etodolac	Diclofenac	Internal
Clotrimazole	Etoposide	Diflunisal	Internal
Clozapine	Fentiazac	Diltiazem	Internal
Codeine	Fluconazole	Dofetilide	Lorazepam
Diphenhydramine	Flucytosine	Etodolac	Midazolam
Disopyramide	Haloperidol	Furosemide	Nifedipine
Flecainide	Imipramine	Glipizide	Oxaprozin
Fluconazole	Ketoconazole	Granisetron	Prazosin
Haloperidol	Loratadine	Internal	Terfenadine
Imipramine	Methotrexate	Internal	Troglitazone
Lidocaine	Metoclopramide	Internal	Warfarin
Metoclopramide	Metoprolol	Imipramine	Verapamil
Metronidazole	Nadolol	Irbesartan	
Mexiletine	Nimesulide	Ketanserin	
Nizatidine	Octreotide	Lorazepam	
Pirenzepine	Pindolol	Methylprednisolone	
Prednisolone	Pravastatin	Metoprolol	
Prednisone	Prazosin	Midazolam	
Procainamide	Promethazine	Nifedipine	
Propafenone	Propranolol	Ondansetron	
Propranolol	Quinidine	Oxaprozin	
Quinidine	Rifabutin	Phenancetin	
Ranitidine	Rifampin	Pindolol	
Risperidone	Sildenafil	Prazosin	
Sotalol	Sulfamethoxazole	Prednisolone	
Sumatriptan	Sulfaphenazole	Quinidine	
Tetracaine	Suprofen	Sildenafil	
Thioridazine	Terbutaline	Theophylline	
Tiotidine	Timolol	Timolol	
Triamterene	Tolbutamide	Troglitazone	
Triflupromazine	Triazolam	Trovafloxacin	
Trimethoprim	Trimethoprim	Warfarin	
	Warfarin	Verapamil	
	Verapamil	Zolpidem	
	Zomepirac		
